# Endemicity response timelines for *Plasmodium falciparum *elimination

**DOI:** 10.1186/1475-2875-8-87

**Published:** 2009-04-30

**Authors:** David L Smith, Simon I Hay

**Affiliations:** 1 Department of Zoology and Emerging Pathogens Institute, University of Florida, Gainesville, Florida, USA; 2 Spatial Ecology and Epidemiology Group, Tinbergen Building, Department of Zoology, University of Oxford, South Parks Road, Oxford, OX1 3PS, UK

## Abstract

**Background:**

The scaling up of malaria control and renewed calls for malaria eradication have raised interest in defining timelines for changes in malaria endemicity.

**Methods:**

The epidemiological theory for the decline in the *Plasmodium falciparum *parasite rate (*Pf*PR, the prevalence of infection) following intervention was critically reviewed and where necessary extended to consider superinfection, heterogeneous biting, and aging infections. Timelines for malaria control and elimination under different levels of intervention were then established using a wide range of candidate mathematical models. Analysis focused on the timelines from baseline to 1% and from 1% through the final stages of elimination.

**Results:**

The Ross-Macdonald model, which ignores superinfection, was used for planning during the Global Malaria Eradication Programme (GMEP). In models that consider superinfection, *Pf*PR takes two to three years longer to reach 1% starting from a hyperendemic baseline, consistent with one of the few large-scale malaria control trials conducted in an African population with hyperendemic malaria. The time to elimination depends fundamentally upon the extent to which malaria transmission is interrupted and the size of the human population modelled. When the *Pf*PR drops below 1%, almost all models predict similar and proportional declines in *Pf*PR in consecutive years from 1% through to elimination and that the waiting time to reduce *Pf*PR from 10% to 1% and from 1% to 0.1% are approximately equal, but the decay rate can increase over time if infections senesce.

**Conclusion:**

The theory described herein provides simple "rules of thumb" and likely time horizons for the impact of interventions for control and elimination. Starting from a hyperendemic baseline, the GMEP planning timelines, which were based on the Ross-Macdonald model with completely interrupted transmission, were inappropriate for setting endemicity timelines and they represent the most optimistic scenario for places with lower endemicity. Basic timelines from *Pf*PR of 1% through elimination depend on population size and low-level transmission. These models provide a theoretical basis that can be further tailored to specific control and elimination scenarios.

## Background

The Roll Back Malaria Partnership (RBM) recently called for universal coverage with vector control and effective drugs by 2010 [1], and major international agencies are realigning their short-term goals and activities to achieve the long-term goal of global malaria eradication [2-4]. Many countries are now contemplating malaria elimination, so there is a need to define plausible timelines for planning. Much of the previous experience in setting malaria elimination timelines comes from the Global Malaria Eradication Programme (GMEP), which was launched in 1955 and implemented in four phases called planning, attack, consolidation, and maintenance [5,6]. The attack phase was based on three to four years of indoor spraying with residual insecticides to completely interrupt transmission; success was judged by several criteria, one of which was that by the end of spraying, the *Plasmodium falciparum *parasite rate (*Pf*PR, the prevalence of malaria infection) was reduced to less than 1% of the pre-control baseline [7]. The GMEP prioritized this short duration attack phase to reduce the opportunity for the evolution of insecticide resistance in *Anopheles *vectors. During the GMEP, expected *Pf*PR declines within the attack phase were based on the Ross-Macdonald model [7].

The end of a successful GMEP attack phase marked the beginning of a new control phase, called consolidation, defined in part by new requirements for monitoring and evaluation; when *Pf*PR drops below 1%, parasite surveys become an inefficient way of sampling malaria and measuring progress [8]. The focus of malaria control programmes during consolidation shifted to developing health and surveillance systems, identifying and eliminating residual transmission foci and preparing countries for malaria free status certification [9]. While the GMEP established well-defined timelines for the attack phase, timelines for the consolidation phase were not outlined [8].

**Figure 1:**
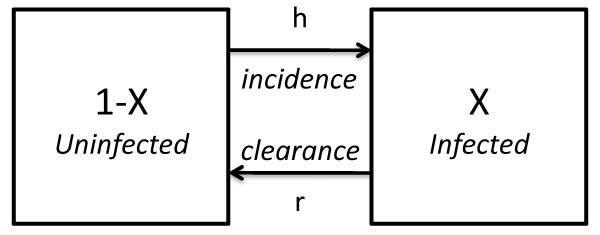
**A diagram of human infection dynamics in the Ross-Macdonald model, which was used for planning during the GMEP**. Changes in the fraction of infected humans, denoted *X*, were described by two simple rules. The incidence rate of new infections or "force of infection", denoted *h*, describes the *per capita *rate that uninfected humans would become infected. Clearance of existing infections, the *per capita *rate that infected humans lose infections, was denoted *r*. Human infection dynamics are described by an equation:  = *h*(1 - *X*) - *rX*, where the superdot represents the derivative with respect to time.

By 1964, several GMEP control initiatives outside of Africa had successfully reduced *Pf*PR below 1% after three to four years of spraying [7], which provided some validation for the GMEP model and timelines, but there were fewer successes in Africa [10]. Before GMEP, there had been a polarizing debate among malaria experts about the feasibility of malaria control and elimination in hyperendemic Africa [11], which is defined by *Pf*PR in children aged 2–10 greater than 50% [12]. In Kenya and Tanzania, the Pare-Taveta Malaria Scheme was launched in 1955 to help address many points of contention [11]. The indoor residual spraying campaign in Pare and Taveta lasted for three and a half years during which the *Pf*PR remained high initially, but then fell from above 60% to around 5% in older children [13,14] (Figure 1). The results of the insecticide spraying when evaluated using traditional criteria were not thought to have demonstrated complete interruption of transmission [7]. Direct estimates suggested the basic reproductive number for malaria [15] had been reduced to below one [13], however, and that the decay rates were consistent with a reproductive number under control of 0.7 [7].

A result of intense exposure to malaria in populations with hyperendemic malaria, such as those in Pare-Taveta, is that new infections accumulate on top of uncleared infections: a phenomenon called "superinfection" [16]. These complex infections are comprised of multiple founding parasite genotypes from multiple infectious bites, and the number of distinct founding parasite genotypes is called the multiplicity of infection (MOI). Theory [16-19] and empirical studies [20] have suggested that infections with high MOI take longer to clear. The Ross-MacDonald model used by the GMEP assumed that infections were simple (the MOI was at most one) and, therefore, may have set inappropriate timelines for changing endemicity from a hyperendemic baseline.

Superinfection had been discussed and modelled before the GMEP [16,21], and several additional models have been developed since [18,22,23]. These alternative models had not been used to re-evaluate response timelines to intervention in areas where superinfection would have been common, such as Pare-Taveta. It is clear that the same theory can be also be used for predicting the results of scaling up of malaria interventions today [1].

In this study, the theoretical basis for estimating endemicity responses following intensive control across the malaria transmission spectrum is critically reviewed and extended. A range of simple mathematical models of malaria transmission and parasite clearance were investigated, to describe likely timelines for the changing *Pf*PR in hyperendemic areas from baseline to 1%. The theory is further extended to establishing basic expectations about the timelines for the consolidation phase, the likely timelines for the changes in *Pf*PR from 1% to elimination.

## Methods

The timelines for changing *Pf*PR following malaria control without mass drug administration are determined by the waiting times to clear untreated human infections. The Ross-Macdonald model, on which GMEP planning was based, was used as a starting point for analysis. To evaluate whether the Ross-Macdonald forms a good basis for planning across the endemicity spectrum and during all phases of malaria control and elimination, changes in *Pf*PR were simulated and compared using several other models of malaria infections. These models are described briefly in the following sections. A more detailed mathematical description of the models is found in Additional File 1.

### Clearance rates

The waiting time to clear a simple infection is an important parameter in these models. Estimates of the waiting time to clear an infection come from several different sources. One important source was data from the malaria therapy of neurosyphilis patients, which estimated an average duration of 220 days [24]. The 200-day waiting time was also consistent with an older study in Puerto Rico [25], and with recent studies that compared models and estimated waiting time to clear infections of 150 days in northern Ghana [26].

In these analyses, the expected changes in *Pf*PR were examined under the assumption that the average duration of an infection is 200 days. This value was fixed by the empirical evidence outlined above and numerous studies conducted during the GMEP, where transmission was considered by many criteria to be completely interrupted and the documented declines in *Pf*PR were broadly consistent with an average duration of infection and maximum rate of decline of about 200 days [7].

### Ross-Macdonald

The Ross-Macdonald model assumed that people were either infected or not, and that once infected, infections would clear at a constant per-capita rate, *r*. The assumption applied regardless of how long a person had already been infected or whether the person had acquired multiple infections. The model thus implies that the waiting time to lose an infection is exponentially distributed with a mean waiting time of *1/r *days. During the GMEP, *r *was assumed to be 0.5% per day, which implied an average waiting time to clear of 200 days. Figure 1 shows a diagram of the human infection dynamics in the Ross-Macdonald model.

### Queuing models

A challenge to the Ross-Macdonald model is that it does not consider the time to clear malaria when superinfection occurs. A simple formula to describe the waiting time to clear complex infections while transmission continues was derived during the Garki Project [17], but the formula converges rapidly to the Ross-Macdonald model if vector-control measures reduce transmission (Additional File 1).

**Figure 2:**
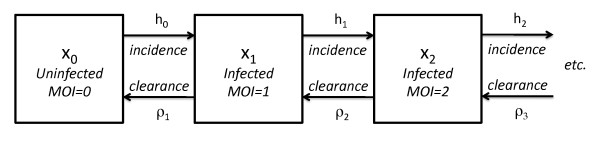
**A diagram of the queuing models, which extend the Ross-Macdonald model by tracking changes in the MOI**. The fraction of the whole population with MOI of *m*, denoted *x*_*m*_, changes when new infections occur or when existing infections clear, and in any short interval of time, one individual's MOI increments or decrements by one [18]. The rate that new infections arise may depend on MOI, denoted *h*_*m*_. The rate of loss may depend on MOI, denoted *ρ*_*m*_, and *ρ*_1 _= *r *denotes the rate a simple infection is lost. Changes in the fraction of the population that is uninfected are described by an equation:  = -*h*_0_*x*_0 _+ *rx*_1_. Changes in the fraction of people who are already infected with a given MOI are described by a set of equations:  = -*h*_*m*_*x*_*m *_+ *h*_*m*-1_*x*_*m*-1 _- *ρ*_*m*_*x*_*m *_+ *ρ*_*m*+1_*x*_*m*+1_. These equations describe a family of queuing models: each queuing model makes different assumptions about infection and clearance. In "infinite strain" models, *h*_*m *_= *h*, and in "finite strain" models where *M *denotes the maximum number of types, *h*_*m *_= *h*(1-*m*/*M*). Models considered parasite types that cleared independently, or with competition or facilitation. For independent clearance, individual types were unaffected by concurrent infection with other parasite types, so *ρ*_*m *_= *rm*. Competition and facilitation were modelled by letting *ρ*_*m *_= *rm*^σ^, where *σ *>1 described competition and *σ *<1 implies facilitation. Compared with independent clearance, per-strain clearance rates are faster with competition (i.e. *ρ*_*m*_> *rm*), and slower with facilitation (i.e. *ρ*_*m*_<*rm*). Clearance rates per person increase with MOI in all the models; if no new infections occurred, the expected waiting time to lose all the existing infections would be the sum of times to progressively decrement MOI: 1/*r*+1/ρ_2_+1/ρ_3_+...+1/ρ_*m*_.

To describe the timelines for changing MOI when transmission is interrupted, it is necessary to track changes in the full distribution of MOI. Superinfection and dynamic changes in *Pf*PR were, therefore, simulated using a family of queuing models [18]. Queuing models were extended here to consider other biological scenarios, including finite or infinite "strains," and infections that cleared independently, or with competition that would increase clearance rates of parasite types, or with facilitation that would slow down clearance rates of parasite types (Figure 2). The queuing model was further extended to include heterogeneous biting; a queuing model described the dynamics of MOI in each human population stratum, defined by their relative rate of exposure.

### Stochasticity

The Ross-Macdonald model and the queuing models are deterministic: nothing happens by chance. Deterministic models are good approximations when the law of large numbers applies. In the approach to malaria elimination, however, as the number of people who are infected becomes small, it is no longer sensible to ignore chance. During the consolidation phase, there must be a point when the emphasis begins to shift from the proportion of a population that is infected to the number infected. *Pf*PR defines the basis for switching malaria control phases, in part because of the impractically large sample sizes required to use changes in *Pf*PR as a measure of progress. When *Pf*PR is 1%, the number of people who remain infected is proportional to the population size. A *Pf*PR of 1% means, for example, that one person is infected in a population of 100 people, but that ten thousand people would be infected in a population of a million. Starting from a *Pf*PR of 1%, the waiting time to elimination is, therefore, longer in larger populations because there are more people who remain infected. To consider stochasticity and the effect of population size on the time to elimination, stochastic analogues of the Ross-Macdonald model and the queuing models were also developed and the changes in *Pf*PR were simulated in populations of various sizes.

### Stage-structured models

The Ross-Macdonald model and the queuing models consider the waiting time to clear an infection without regard to the age of the infection. In the Ross-Macdonald model, for example, a person who has been infected for just one week and a person who has already been infected for 200 days are equally likely to remain infected for 200 more days. This may not be realistic if clearance rates change with the age of the infection. Stage-structured models are a useful way to model infections that clear faster or slower as they age [27], but it is computationally difficult to model stage-structure and superinfection deterministically (Additional File 1). Aging infections were modelled in stochastic models with superinfection, under the assumption that infections clear independently. An extreme version of this model was tested here in which infections progressed through *n *equal "stages," and were cleared from the last stage. This is called senescence because infection rates increase as infections age [27].

### R_0 _and vector dynamics

Mosquito infection dynamics and the force of infection were simulated in a minimal mosquito model (Additional File 1). In all these models, the intensity of transmission by mosquitoes in the absence of control was determined by the basic reproductive number, *R*_0_. The intensity of transmission under control was determined by the controlled basic reproductive number, *R*_*C*_. The two terms are directly analogous in every way but one; whereas *R*_0 _is only defined in an environment that lacks control, *R*_*C *_describes potential transmission in a population with some level of control. In the simulations, *R*_0 _sets the baseline, which determines the *Pf*PR at the start of a simulation. In models with superinfection, *R*_0 _also sets the equilibrium distribution of MOI. *R*_*C *_determines the rate of decline in *Pf*PR and the waiting time to elimination from that baseline. Transmission would be interrupted eventually if malaria elimination were the endpoint in the absence of imported malaria (*R*_*C *_< 1), but it would be completely and immediately interrupted if *R*_*C *_= 0.

## Results

**Figure 3:**
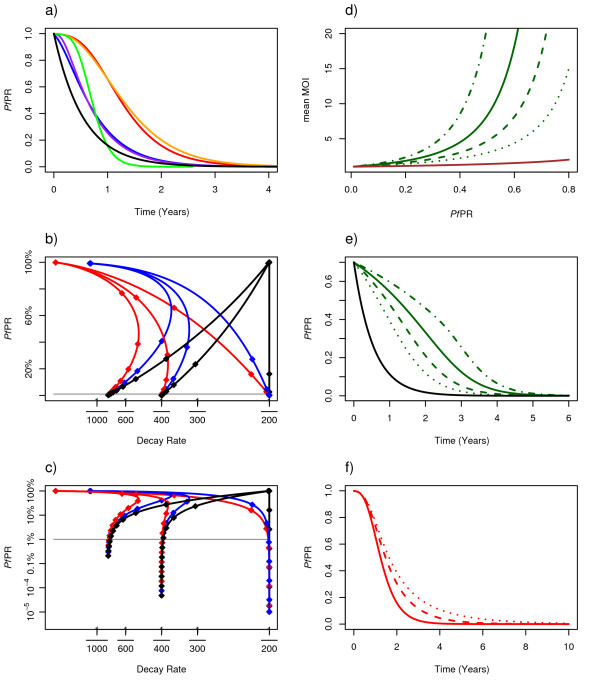
**a) The decline in *Pf*PR for six different models with completely interrupted transmission: the Ross-Macdonald model (black), infinite strains, homogeneous biting, and independent clearance (red), two strains, homogeneous biting and independent clearance (blue), infinite strains, homogeneous biting and competition (purple), four strains with homogeneous biting and facilitation (orange), and senescing infections (green)**. b)The daily decay rates for several models have been plotted as a function of declining *Pf*PR, which is plotted on the vertical axis. The decay rate is the log of the PR ratio on consecutive days. Diamonds along these trajectories are plotted one year apart. The models have the same colors as in the panel above: the Ross-Macdonald model (black), infinite strains, homogeneous biting and independent clearance (red), and two strains, homogeneous biting and independent clearance (blue). The trajectories have also been plotted for completely interrupted transmission as above and for two larger values of *R*_*C *_(0.5, and 0.75), which reach different asymptotic decay rates. c)The same graph has been re-plotted on a logarithmic scale for *Pf*PR to show that the decay rates remain constant at *r*(*R*_*C*_-1) after *Pf*PR is below approximately 1%. d)The relationship between baseline *Pf*PR and the predicted MOI for the model with heterogeneous biting at various levels: α = 2, dotted; α = 3, dashed; α = 4.2, solid; α = 6, dash-dot; and homogeneous biting, red. The more aggregated the biting, the higher the average MOI for the same *Pf*PR. e)The *Pf*PR over time for the same models as panel dand completely interrupted transmission starting from a baseline *Pf*PR of 70%, and compared with the Ross-Macdonald model (black). f)The decline in *Pf*PR for a model with infinite strains, homogeneous biting, and independent clearance, but with different values of *R*_*C *_(solid, *R*_*C *_= 0; dashed, *R*_*C *_= 0.5; and dotted, *R*_*C *_= 0.75).

The results focus on the *Pf*PR timelines and decay rates when the endpoint was elimination. The *Pf*PR over time was simulated numerically in all these models and compared plotting the *Pf*PR over time. Another useful way of comparing models was to plot the decay rates versus *Pf*PR (Figure 3). The daily decay rate is the logarithm of the daily change in *Pf*PR, and over any interval, the decay rate is the logarithm of the change in *Pf*PR divided by the length of the interval.

### PfPR from baseline to 1%

In all the queuing models, the waiting time to clear malaria increases with MOI; it is the sum of waiting times to clear a simple infection (*1/r*) plus the time to progressively decrement MOI. With independent clearance, for example, the expected waiting time to clear an infection with *m *types was therefore *1/r + 1/2r + 1/3r + ... + 1/mr*. Queuing models with competition, independent clearance, or facilitation each made quantitatively different predictions about the decline of *Pf*PR from baseline to 1% (Figure 3a). Some model identifiability problems were obvious. A model with four strains and facilitation was very similar to a model with infinite strains and independent clearance, for example, and a model with infinite strains and competition was similar to a model with two strains and independent clearance. As a general rule, models with a large number of strains or facilitation had longer timelines, while models with a small number of strains or competition had shorter timelines.

The queuing models predicted different rates and patterns of decline than the Ross-Macdonald model (Figure 3a, b, c). In the Ross-Macdonald model, the decay rate in *Pf*PR was initially faster than in models with superinfection, but the *Pf*PR decay rate subsequently declined. In queuing models in hyperendemic areas, the decay rate was initially slow as MOI declined, but after average MOI had declined to approximately one, *Pf*PR decay rates then followed the same pattern as the Ross-Macdonald model. The initial rate of decline was related to the initial distribution of MOI. Each one of the queuing models made a different prediction about the distribution of MOI in relation to baseline *Pf*PR, and heterogeneous biting strongly influenced this relationship (Figure 3d). When biting was more aggregated on a few individuals, the average MOI at a given level of endemicity was higher, and the initial rate of decline was slower (Figure 3e). The average MOI increased with *Pf*PR, but it remained close to one until malaria was hyperendemic, when it began to increase sharply (Figure 3f). The transition from an area with predominantly simple infections to one with predominantly complex infections was related to the degree of heterogeneous biting; for the same *Pf*PR, higher *R*_0 _and higher MOI were correlated. The queuing models did not differ substantially from the Ross-Macdonald model at low endemicity when MOI is typically close to one, which is consistent with the GMEP experience outside Africa [7]. Because of the slow initial rate of decline, the waiting time to reach a *Pf*PR of 1%, starting from a hyperendemic baseline, was two to three years longer than the Ross-Macdonald model.

The initial rate of decline differed substantially among the models, and it was also affected by the degree of biting heterogeneity, but it was not affected by *R*_*C*_. Holding all else but *R*_*C *_equal, the differences in *Pf*PR over time were not apparent until *Pf*PR had fallen (Figure 3f). In other words, the model determined the initial rate of decline, but *R*_*C *_determined the asymptotic rate of decline. In models that ignored the age of the infection, when *R*_*C *_= 0, the fastest decay rate possible was *r*. In other words, the GMEP planning timelines, which used the Ross-Macdonald model with completely interrupted transmission, described the most optimistic timelines for changes in *Pf*PR from baseline to 1%.

Table 1 reports the waiting time to reach a *Pf*PR of 1% in the infinite queuing model with heterogeneous biting. This model provides a good empirical fit to data describing the entomological inoculation rate and *Pf*PR in African children [28], and it also has other empirical support [29]. The values are shown for *Pf*PR baselines ranging from 5% up to 80%, and for a range of *R*_*C *_values. Also shown are the waiting times to reach *Pf*PR of 1% for *R*_*C *_= 1.05, when the endpoint *Pf*PR is 0.7%.

**Table 1:** The expected waiting time (in years) to reach *Pf*PR of 1% from a range of different baselines.

	Controlled Reproductive Number, *R*_*C*_					
Baseline *Pf*PR	*R*_*C *_= 0	*R*_*C *_= 0.5	*R*_*C *_= 0.75	*R*_*C *_= 0.9	*R*_*C *_= 1	*R*_*C *_= 1.05
5%	0.9	1.6	2.5	3.8	6.2	9.4
10%	1.4	2.2	3.4	5.0	7.6	10.8
15%	1.8	2.6	3.8	5.5	8.2	11.5
20%	2.0	3.0	4.2	5.9	8.5	11.9
25%	2.2	3.2	4.5	6.1	8.8	12.2
30%	2.5	3.5	4.7	6.4	9.1	12.5
35%	2.7	3.7	5.0	6.7	9.3	12.7
40%	2.9	3.9	5.2	6.9	9.6	12.9
45%	3.1	4.1	5.4	7.1	9.8	13.2
50%	3.3	4.4	5.7	7.4	10.1	13.4
55%	3.6	4.7	5.9	7.6	10.3	13.7
60%	3.9	4.9	6.2	7.9	10.6	14.0
65%	4.2	5.2	6.5	8.2	10.9	14.3
70%	4.5	5.6	6.9	8.6	11.3	14.6
75%	4.9	5.8	7.1	8.8	11.5	14.8
80%	5.2	6.0	7.2	8.9	11.7	15.0

These values were computed using the queuing model with heterogeneous biting, infinite strains and independent clearance. The columns represent waiting times for different values of the controlled reproductive number, *R*_*C*_, that show representative waiting times from zero up to 1.05. The projections assume a sudden reduction from *R*_0 _to *R*_*C*_. Longer timelines are expected if *R*_*C *_is reduced gradually.

### From 1% to elimination

After *Pf*PR falls below 1%, the decay rate of *Pf*PR in all the models, except for those with senescing infections, was approximately *r*(*R*_*C*_-1) because superinfection is rare at very low endemicity (Figure 3, bottom). In the deterministic Ross-Macdonald model, declines in *Pf*PR from 1% through to elimination are approximated by the formula (SI):

(1)

With a 200 day waiting time to clear infections, as the GMEP model assumed, completely interrupted transmission would result in an 84% proportional reduction in *Pf*PR each year. Compared with completely interrupted transmission, *Pf*PR declines half as fast at *R*_*C *_= 0.5, and a fourth as fast at *R*_*C *_= 0.75. The model predicts that the ratio of *Pf*PR in consecutive years remains constant through elimination; the waiting time to reduce *Pf*PR from 10% to 1% is approximately the same as the waiting time to reduce *Pf*PR from 1% to 0.1%, or from 1,000 infected individuals to 100.

The expected waiting time to elimination, *T*_*E*_, can be approximated using Eq. 1 as the time when only one person in the population is infected, or in a population of size *H*, when *Pf*PR is less than *1/H*:

(2)

The larger *H*, therefore, the longer it takes to achieve elimination. In a population of 1,000 humans with completely interrupted transmission (i.e. *R*_*C *_= 0), It would take 2.5 years to eliminate malaria from a population of 1,000 people, but 5 years from a population of 100,000. The waiting times also depend on *R*_*C*_. Compared with completely interrupted transmission, the time to elimination takes twice as long if *R*_*C *_= 0.5 and four times as long if *R*_*C *_= 0.75. Sample values are shown in Table 2.

**Table 2:** The expected waiting time (in years) to elimination starting from *Pf*PR = 1%.

	Population size			
Basic reproductive number under control (*R*_*C*_)	H = 10^3^	H = 10^4^	H = 10^5^	H = 10^6^
0.00	2.5	3.8	5.0	6.3
0.05	2.7	4.0	5.3	6.6
0.10	2.8	4.2	5.6	7.0
0.15	3.0	4.5	5.9	7.4
0.20	3.2	4.7	6.3	7.9
0.25	3.4	5.0	6.7	8.4
0.30	3.6	5.4	7.2	9.0
0.35	3.9	5.8	7.8	9.7
0.40	4.2	6.3	8.4	10.5
0.45	4.6	6.9	9.2	11.5
0.50	5.0	7.6	10.1	12.6
0.55	5.6	8.4	11.2	14.0
0.60	6.3	9.5	12.6	15.8
0.65	7.2	10.8	14.4	18.0
0.70	8.4	12.6	16.8	21.0
0.75	10.1	15.1	20.2	25.2
0.80	12.6	18.9	25.2	31.5
0.85	16.8	25.2	33.6	42.1
0.90	25.2	37.9	50.5	63.1

The columns represent waiting times for different human population sizes ranging in size from one thousand to one million. The rows show the waiting times for *R*_*C *_values from zero up to 0.9. These values were computed using Eqn. 6, which agrees with the median time to elimination for the simulations when *R*_*C *_= 0.5, but when *R*_*C *_= 0.75, the median extinction times were slightly shorter (Figure 2).

The waiting time to elimination was simulated in the stochastic analogue of the models starting from a *Pf*PR of 1%; using *R*_*C *_as an index of ongoing transmission, all of the queuing models produced similar results, including those with heterogeneous biting (see Additional File 1). As the number of people who were infected becomes small (<1,000), stochasticity introduces noticeable fluctuation in the *Pf*PR and this affects the time to elimination.

**Figure 4:**
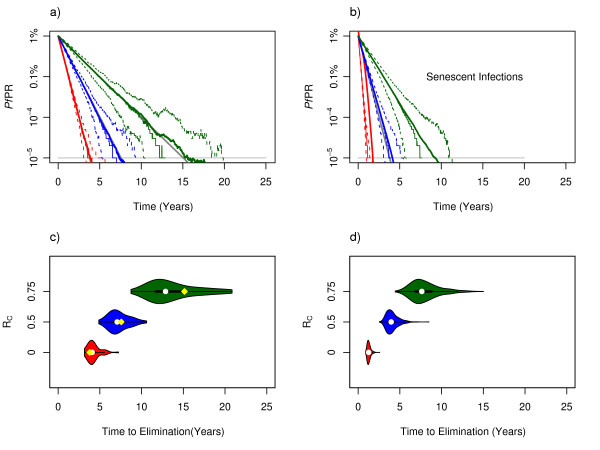
**a) Using a stochastic model, *Pf*PR was simulated over time starting from 1% and following through to elimination (defined as when no one remains infected) in a population of 100,000 people using the Ross-Macdonald model for *R*_*C *_= 0 (red), *R*_*C *_= 0.5 (blue), and *R*_*C *_= 0.75 (green)**. The solid line shows the median *Pf*PR over time from the ensemble, the dashed lines show the 5^th ^and 95^th ^quantiles, and the solid grey lines (sometimes covered by the median *Pf*PR) show the same results for the deterministic models. b)The same plots for ensembles of stochastic simulations using *Gamma *distributed infectious periods (n = 4), for which infections effectively "senesce". The grey line (under the solid blue line) shows the Ross-Macdonald model for completely interrupted transmission. c, d)For the same ensemble of 500 simulations corresponding to the panels above, these violin plots show a kernel density plot for the distribution of extinction times, in years. The white dots show the median time to extinction, the thick black lines show the inter-quartile range. In the left-hand panel, the yellow dots show the predicted values from Equation 6, which are fairly close to the average waiting time to reach *1/H *from the ensemble of simulations.

The average proportional reductions in *Pf*PR from the ensemble of simulations tracked the deterministic models, but there was noticeable variability when populations became small. The waiting times to elimination were, therefore, extremely variable. Similar results were predicted by all of the models, except those with senescent infections, which produced much shorter and less variable waiting times to elimination when transmission was completely interrupted (Figure 4). Again, there were model identifiability issues; a model with senescence and *R*_*C *_= 0.5 was similar to a model with no senescence and completely interrupted transmission (Figure 3b). Without senescent infections, the sharpest annual decline in *Pf*PR that would be expected is 84%, but for models with senescent infections, the *Pf*PR decay rate would steadily increase and much faster declines are possible.

### The Pare-Taveta malaria scheme

Because of model identifiability issues and some uncertainty about the underlying biology of *P. falciparum *transmission, it is impossible to make strong conclusions about whether malaria transmission had been interrupted in Pare-Taveta based upon only the initial decay rate in *Pf*PR. Other evidence suggested that transmission had not been interrupted, especially malaria infections in infants who had never travelled outside the sprayed area [13,14].

**Figure 5:**
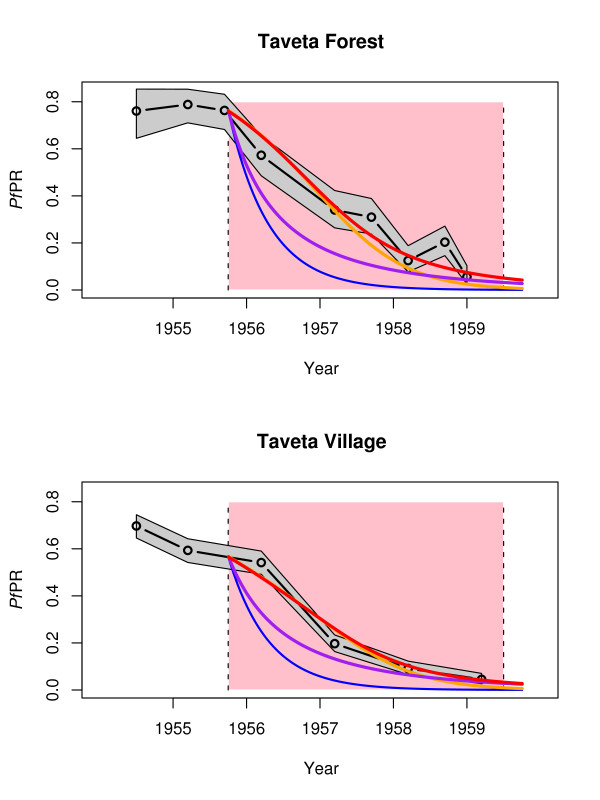
**The parasite rate in two parts of Taveta **[14]**: the forest (top) and the village (bottom)**. The data track the *Pf*PR in 2–10 year olds starting from 1954 and extending through 1959 – the grey shows the binomial confidence intervals by the exact test. Spraying started late in 1955 with effects that extended through 1959 (shown in pink). The blue line shows the Ross-Macdonald model with *R*_*C *_= 0 that was used for planning during the GMEP, the purple line shows the Ross-Macdonald model with *R*_*C *_= 0.7, the orange and red line shows the infinite queuing model with heterogeneous biting (α = 3) and *R*_*C *_= 0 (orange) and with *R*_*C *_= 0.7 (red).

The initial decay rates in Pare and Taveta, where malaria was hyperendemic at the baseline, were slow initially and then increased, consistent with models of superinfection (Figure 5). Exponential decay models also provide a good statistical fit to the data, but the rate is difficult to interpret as a measure of ongoing transmission in the context of a malaria transmission model [30]. One model of interest is the queuing model with heterogeneous biting and independent clearance, which provides a good fit to estimates of the entomological inoculation rate and *Pf*PR, and the parameter estimates from the model fitting were consistent with direct observations [28]. Because of the low number of data points, however, there is not sufficient statistical power to test the models with any degree of confidence. Nevertheless, superinfection does occur in hyperendemic areas, and the models provide a better visual fit to the observed patterns [29].

## Discussion

The Ross-Macdonald model was used during the GMEP for planning endemicity response timelines during the attack phase, when *Pf*PR was reduced from the pre-control baseline to 1%. A comparison of several different models suggests that the Ross-Macdonald model remains useful today in planning for the responses to massive scaling-up of malaria control, but with two important caveats; the GMEP timelines represent the most optimistic scenario in areas where superinfection is uncommon, and in hyperendemic areas where superinfection is common, the GMEP timelines are unrealistically fast. Queuing models suggest that, because of superinfection, the initial decay rates are very slow in hyperendemic areas. As *Pf*PR declines and MOI approaches one, after two to three years of intensive control, the decay rates will resemble places that started from a lower baseline. This pattern was consistent with the response to malaria control observed in Pare-Taveta. Reasonable "attack" phases to bring *Pf*PR to below 1% could last two to three years longer than the GMEP timelines. During the GMEP, the three to four year response timelines were inadequate for hyperendemic Africa, including Pare-Taveta. Also of note is that interventions during the Garki Project lasted only 18 months [31].

These models also generate useful timelines for the consolidation phase. Many studies have suggested that *R*_*C *_< 1 represents a threshold for elimination, but this analysis suggests that the timelines for control and elimination are extremely long unless *R*_*C *_is reduced substantially below one. High levels of control must also be sustained for several years after *Pf*PR falls below 1%. The waiting time to fall from 10% to 1% is the same as the waiting time to fall from 1% to 0.1% or from 1,000 infected individuals to 100 infected individuals. After reducing *Pf*PR below 1%, therefore, the waiting time to complete elimination can be longer than the waiting time to reach 1%, depending on the population size and the degree to which malaria transmission is interrupted.

An important recommendation is that malaria control planning timelines for consolidation after *Pf*PR drops below 1% should be based on the number of people who are infected, not the *Pf*PR. Large populations must wait longer than small populations to achieve elimination because when *Pf*PR is 1%, more people remain infected in the larger population. After reaching a low endemicity of 1%, the waiting time to elimination ranges from 2.5 additional years in a population of a thousand humans to 6.3 additional years in a population of a million, assuming transmission is completely and immediately interrupted. When there is some low level transmission, populations of a million people can reasonably expect to wait a decade or more until the endemic reservoir of parasites has cleared.

These timelines are largely based on models that ignore the age of malaria infections. If infections do "senesce" such that older infections clear faster than new ones, the time to elimination would be much shorter. There has been some mixed evidence for senescence [29,32], but *Pf*PR decay rates that are broadly consistent with non-senescing infections were found often during GMEP experiences [7]. In the documentation of successful attacks during the GMEP, decay rates were found that were faster than the predicted 84% maximum decline, but only when programmes used drugs, or when *Pf*PR was very low and sampling error was an important concern [7]. The GMEP studies were also generally of short duration (three to four years), so that senescence might not have become apparent in the data. When there is some ongoing low-level transmission on the way to elimination, infections in the population will be a mix of young and old infections, and senescence may not be important or apparent. Because of uncertainty about senescence, however, projections based on the Ross-Macdonald model should be considered a worst-case scenario for the consolidation phase, because shorter elimination timelines can be expected if infections senesce.

There is some uncertainty about the waiting time to clear an infection, and the timelines described here would change if the duration of an infection were actually closer to 150 or 250 days. This analysis has focused instead on comparing models, and it demonstrated that the same patterns could arise from differences in either *r*, in *R*_*C*_, or in the model. A basis exists for predicting *R*_0 _based on *Pf*PR and *Pf*EIR [28,33], and there is also a basis for predicting *R*_*C *_in relation to usage of insecticide treated nets [34,35], so it is possible to develop a predictive theory for malaria control endpoints and timelines. Regardless of the uncertainty, most models give broadly similar results when transmission intensity is reduced below the threshold for elimination, but not completely interrupted (i.e. when *R*_*c *_< 1).

One way to resolve the identifiability issues is to design studies that concurrently estimate *r*, *Pf*PR, MOI, and *R*_*C*_. The analysis thus suggests that a field-estimate of MOI could be an important complement to *Pf*PR. After fixing *Pf*PR, biting heterogeneity, *R*_0_, and the time to reach *Pf*PR of 1% all increase with MOI. A gap currently exists, however, between the notion of MOI in these models and MOI in studies.

MOI in the models is based on the notion of a parasite "brood," the number of founding parasite genotypes. Superinfection can also arise if a person is simultaneously bitten by many infectious mosquitoes [36], or if a bite transmits several parasite genotypes. The problems of defining broods and relating these to empirical estimates of MOI are complicated by parasite populations that are highly genetically polymorphic and infections that are highly variable – parasite population densities and frequencies can fluctuate by several orders of magnitude over short periods of time. Similar counting problems arise in ecology, where there has been a long-standing interest in estimating species diversity [37]. Ecologist measure diversity in two ways: richness is the total number of different entities present, and evenness is the probability that any two random individuals would have the same type. Sequencing methods that sample random parasites tend to measure evenness, not richness, because they resample common types. Sequencing methods that identify genetic polymorphisms can't infer the genotypes of individual parasites, which sets an upper limit on the ability to estimate MOI. MOI in the models is based on the notion of richness, so the relevance of existing MOI estimates not clear. If MOI could be developed as a standardized and interpretable field-measure of transmission, it would represent a highly useful complement to *Pf*PR as a basis for setting timelines.

Timelines for elimination can be advanced with antimalarial drugs. Mass drug administration can rapidly reduce the number of infected people and advance the timelines for elimination, but the expense and risks may not be justified since the declines would happen regardless. The use of drugs to cure malaria infections can prevent ongoing transmission, reduce *R*_*C *_and speed up elimination; other models suggest that using drugs for treating clinical malaria controls transmission better in low endemicity settings [27]. Active case detection to find, treat and cure nearby asymptomatic infections can speed up progress towards elimination. Prompt treatment of a high fraction of clinical malaria, case-based investigations, and active case detection become increasingly important for reducing ongoing transmission from imported malaria in the end phases of consolidation and after malaria has been eliminated [38], so implementing such policies during the consolidation phase provides an opportunity to train and build capacity.

This modelling framework was designed to establish basic expectations and a template that can be tailored to local situations. Planning for malaria elimination must also consider the risk of imported malaria and ongoing transmission from imported cases. This is of great concern during elimination campaigns, and it would continue to be a concern after achieving elimination, but it is a separate issue from the initial goal of eliminating the endemic reservoir. Other important considerations are natural fluctuations in mosquito population densities, changes in the coverage levels or effectiveness of various interventions, and the spatial structure of populations. These factors would lead to quantitative changes and increased variability in the expected waiting times to elimination, but so long as there is not a temporal trend in these factors, the basic expectations established here would still be useful. Given the large number of factors and possible permutations of those factors, it would be impractical to consider every scenario prospectively. Expansion of the basic models may be best done in case studies, which can consider these other factors in country-specific malaria elimination plans.

Several models were used to evaluate decay rates in *Pf*PR in response to malaria control; the analysis demonstrates that many different plausible models produce very similar patterns. The timelines described here can help to establish reasonable planning horizons for countries that are contemplating elimination. A substantial body of further work is required to investigate the most locally appropriate and effective suite of interventions needed to affect that goal. This modelling framework, when combined with contemporary global maps of *P. falciparum *prevalence [39] and summaries of existing intervention coverage [40], provides a basis for setting plausible timelines for changing malaria endemicity in Africa and this is the subject of ongoing work.

## Abbreviations

(GMEP): Global Malaria Eradication Programme; (*Pf*PR): *Plasmodiumfalciparum *parasite rate; (MOI): multiplicity of infection; (*R*_0_): basic reproductive number; (*R*_*C*_): basic reproductive number under control.

## Competing interests

The authors declare that they have no competing interests.

## Authors' contributions

DLS wrote the first draft of the manuscript and outlined the models. SIH contributed to the refining of the models and the writing of the manuscript.

## Supplementary Material

Additional file 1 **Supplementary Online Information: Endemicity response timelines for *Plasmodium falciparum *elimination**. A fuller description and analysis of the mathematical models. Click here for file
