# Supplementary material for: Endemicity response timelines for Plasmodium falciparum elimination
Source: Malar J. 2009 Apr 30;8:87. doi: 10.1186/1475-2875-8-87 (PMC2686731; doi:10.1186/1475-2875-8-87)
Supplement: Additional file 1 — Supplementary Online Information: Endemicity response timelines for Plasmodium falciparum elimination. A fuller description and analysis of the mathematical models. [file 1475-2875-8-87-S1.pdf]

Supplementary Online Information: Endemicity response timelines for *Plasmodium falciparum* elimination

David L. Smith, Simon I. Hay

April 28, 2009

The Ross-Macdonald Model We use the following simple version of the Ross-Macdonald model. The parameters are described in Box #1. For a longer explanation, see [1].

The change in the PR, the prevalence of infection, is given by the equation:

$$\dot{X} = mabZ(1-X) - rX \tag{1}$$

The change in the sporozoite rate, the fraction of the mosquitoes with sporozoites in their salivary glands, is modeled using the equation:

$$\dot{Z} = acX(e^{-g\tau} - Z) - gZ \tag{2}$$

This equation ignores the delays between the time when a mosquito becomes infected and when it becomes infectious. For these two equations, the basic reproductive number is:

$$R_0 = \frac{ma^2bc}{gr}e^{-g\tau} \tag{3}$$

Because the PR changes on the time-scale of about 200 days, and the sporozoite changes quickly, on the time scales of about 10 days, we assume that the sporozoite rate is approximately at its steady state with respect to the PR:

$$\bar{Z} = \frac{acX}{g + acX}e^{-g\tau} \tag{4}$$

We note that the force of infection, denoted h, is defined in these equations to be:

$$h = mab\bar{Z} = \frac{ma^2bcX}{g + acX}e^{-g\tau} = R_0 r \frac{X}{1 + caX/g} \tag{5}$$

Note that when X is low, then,

$$h \approx R_0 r X \tag{6}$$

We can substitute this back into Equation 1 and rewrite that equation in the following way:

$$\dot{X} = \frac{R_0 r X}{1 + csX}(1 - X) - rX \tag{7}$$

where $s = a/g$ is called the stability index, the average number of human bites per mosquito summed over its entire lifespan. We call this Ross-Macdonald with a minimal mosquito model.

In the situation that is most interesting to us, we start at the steady state of Equation 7, and consider the changes in X over time after we reduce transmission from R_0 to $R_C < 1$. When X gets low, this is approximately:

$$\dot{X} \approx r(R_C - 1)X \tag{8}$$

Note that if a fraction ρ of cases are detected and immediately cured, then R_C counts only the $1 - \rho$ infections that were not appropriately treated.

The Queuing Model We have extended an infinite queuing model for superinfection originally published and described in detail by Bailey [2]. Parameter names are explained in Box #1 and other terms are explained in Box #2. These models consider the dynamics of malaria when a single person simultaneously carries multiple malaria types; the number of types is called multiplicity of infection (MOI). The equations track the changes in MOI. Conceptually, a new infection increases the MOI in a single individual from m to $m+1$, and clearance reduces it from $m+1$ to m.

Let x_m denote the fraction of hosts with an MOI of m, so that $\sum_{m=0}^{\infty} x_m = 1$. The parasite rate is $X = 1 - x_0$. Let h_m denote the force of infection for a person that is already infected with m types, and let ρ_m denote the rate that a person

decrements MOI by one. In an infinite strain model, $h_m = h$, and in finite strain models, we set $h_m = h(1 - m/M)$, where M denotes the maximum number of strains. Independent clearance implies that $\rho_m = rm$. A simple way to model competition or facilitation is to let $\rho_m = (rm)^\sigma$, where clearance rates are faster than independent for $\sigma > 1$, and slower than independent for $\sigma < 1$.

The change in the fraction of uninfected hosts is given by the equation:

$$\dot{x}_0 = -h_0 x_0 + r x_1. \tag{9}$$

Changes in MOI are described by a set of coupled ordinary differential equations:

$$\dot{x}_m = -(h_m + \rho_m)x_m + h_{m-1}x_{m-1} + \rho_{m+1}x_{m+1}. \tag{10}$$

The parasite rate is $X = 1 - x_0$.

The dynamics of infection in mosquitoes were, again, simulated using the minimal mosquito model described in Equation 5.

A special case that we consider here assumes that the potential number of broods is effectively infinite and that each brood clears independently of the others: $h_m = h$ and $\rho_m = mr$. For these assumptions, the distribution of MOI is Poisson with mean $\bar{m} = \bar{h}/r$ [2]. At the steady state, the rate that untreated infections spontaneously clear is $h/(e^{h/r} - 1)$ [3], and note that

$$\lim_{h \to 0} h/(e^{h/r} - 1) = r.$$

While the clearance approximation makes sense for evaluating steady state relations, it does not provide a suitable approximation for tracking dynamic changes in MOI. If the force of infection is suddenly reduced, clearance is determined by the distribution of MOI from the baseline steady state, and a full dynamical model is required to simulate changes in the force of infection and the MOI.

Thus, a simpler model for clearance that is similar to the one used during the Garki project [3] is the following:

$$\dot{X} = h(1 - X) - \frac{h}{e^{h/r} - 1} X \tag{11}$$

It has the same steady state as the queuing model:

$$\bar{X} = 1 - e^{-\bar{h}/r} = 1 - e^{-\bar{m}} \tag{12}$$

It is not appropriate for modeling endemicity response timelines in hyperendemic areas following an abrupt reduction in transmission. Following an abrupt decline in vectorial capacity, h drops, and

$$\lim_{h=0} \frac{h}{e^{h/r} - 1} = r$$

, so the clearance rate rapidly follows the assumption of the Ross-Macdonald model.

Multiplicity of Infection and Heterogeneous Biting A queuing model was also formulated for heterogeneous biting. We have coupled the dynamic MOI model with a minimal mosquito model and extended it to consider multiple subpopulations with different biting rates.

Let j subscripts denote a subpopulation with biting weight ω_j that comprises a fraction W_j of the whole population. Let $x_{m,j}$ denote the fraction of that subpopulation with a given MOI, m. Thus, $\sum_m x_{m,j} = 1$, for all j. The changes in the proportion uninfected within the jth population stratum is:

$$\begin{aligned} \dot{x}_{0,j} &= -h_0 x_{0,j} + r x_{1,j} \\ \dot{x}_{m,j} &= -(h_j + \rho_m) x_{m,j} + \rho_{m+1} x_{m+1} + h_j x_{m-1,j}; \end{aligned} \tag{13}$$

The parasite rate is defined to be

$$X = \sum_j W_j (1 - x_{0,j}). \tag{14}$$

The probability that a mosquito becomes infected after biting a human, denoted $\tilde{X}$ and called net infectivity, is given by the formula:

$$\tilde{X} = \sum_j c\omega_j W_j (1 - x_{0,j}). \tag{15}$$

As before, the dynamic of infections in mosquitoes is motivated by the equation:

$$\dot{Z}/g = s\tilde{X}(e^{-g\tau} - Z) - Z \tag{16}$$

and the entomological inoculation rate (EIR) is the product of the human biting rate and the sporozoite rate $\bar{Z}$; it is given by the equation:

$$\mathcal{E} = R_0 \frac{r\tilde{X}}{1 + s\tilde{X}} \tag{17}$$

Here, we take $\tilde{X} = cx$, and we assume that the force of infection is a linear function of EIR: $h_j = \omega_j b\mathcal{E}$.

We assume that biting weights have a *Gamma* distribution, a very general family of distributions, with mean EIR and a squared coefficient of variation of biting rates given by α. Let $P(\mu)$ denote the proportion of the population with MOI of μ. Under these assumptions, the distribution of MOI at the steady state is negative binomial (using the R parameterization):

$$P(\mu) \sim \text{NegBinom}\left(n = \frac{1}{\alpha}, p = \frac{r}{r + b\mathcal{E}\alpha}\right). \tag{18}$$

At the steady state, *Pf*PR is given by the complement of the zero term from the negative binomial:

$$\bar{X}(\alpha) = 1 - \left(1 + \frac{b\mathcal{E}\alpha}{r}\right)^{-1/\alpha} \tag{19}$$

Eq. 19 thus defines a two-parameter family of curves: a special case is the Ross-Macdonald model (for $\alpha = 1$), although the formulas arise from very different assumptions. Eq. 12 is by taking the limit as α approaches zero [4].

When no estimate of *Pf*EIR is available it can be inferred from *Pf*PR by inverting Eq. 19.

$$\mathcal{E}(\bar{X}) = \frac{r}{b\alpha}\left(\left(1 - \bar{X}\right)^{-\alpha} - 1\right) \tag{20}$$

Obviously, the estimate of *Pf*EIR depends strongly upon on the degree of biting heterogeneity, α. These equations give us the distribution of MOI at the baseline, under the assumptions of the model, using the rule:

$$\bar{X}(\alpha) \to \mathcal{E}(\alpha) \to P(\mu|\alpha) \tag{21}$$

We then choose V to simulate transmission at any controlled reproductive number $R_C = bcV(1+\alpha)/r$. We define completely interrupted transmission to be $R_C = 0$, and any value of $R_C < 1$ describes a low-level of transmission that will eventually lead to elimination. All of these quantitative relations are extremely sensitive to α, the parameter that describes heterogeneous biting.

***Pf*PR Timelines for Completely Interrupted Transmission** Timelines for the declines in *Pf*PR were defined classically for interrupted transmission by the formula: $x(t) = x(0)e^{-rt}$, where $r = 1/200$ days. The predictions of that model were generally consistent with surveillance following a successful attack phase with high household coverage rates with indoor residual spraying at low endemicity [5].

With superinfection, infections take longer to clear [3, 2] (Figure 1,2). In the case when transmission is completely interrupted, i.e. when $R_C = 0$, the exact expressions for the declines in *Pf*PR. For a cohort of people that are all infected with μ distinct types, which all clear independently, the proportion that would remain infected over time is given by:

$$x(\mu, t) = 1 - (1 - e^{-rt})^{\mu} \tag{22}$$

If the distribution of types in a population is $P(\mu)$ at the point in time when transmission is interrupted, then the *Pf*PR declines over time, according to the equation:

$$\sum_{\mu} P(\mu)x(\mu, t) \tag{23}$$

When $P(\mu)$ has a negative binomial distribution described above.

***Pf*PR Timelines for Low-Level Transmission** When transmission is reduced, the changes in *Pf*PR are complicated by ongoing transmission, albeit at a reduced level. As a heuristic, the changing time scales for malaria transmission occur on a fast time scale for vectorial capacity, and on a slower time scale for *Pf*PR. In simple terms $\mathcal{E} \approx Vx$, where V denotes vectorial capacity (see Box #2), so

$$\frac{d\mathcal{E}}{dt} \approx x\frac{dV}{dt} + V\frac{dx}{dt}. \tag{24}$$

The changes in V occur on the time scales of mosquito demography, or approximately $10-20$ days. The changes in *Pf*PR occur on the time-scale of human infections, or around $200-700$ days, depending on the baseline endemicity (Figure 1,2).

Changes in *Pf*PR were found by numerically solving the ordinary differential equations. For numerical simulations, it is necessary to pick a finite number of biting classes and a maximum MOI.To do this, we established a baseline test case by comparing the exact solution for different maximum MOI values. Biting weights were drawn using the following algorithm in R:

```
biting.wts = function(H, alpha=4.2) {
  h = c(1:(H-1))/H
  qh = qgamma(h, shape = 1/alpha, scale = alpha)
  totbit = function(x,alpha=4.2){
    integrand = function(s){
      s*dgamma(s, shape = 1/alpha, scale = alpha)}
    integrate(integrand, 0, x)$value }
  perc = c(0,sapply(qh,totbit,alpha=alpha),1)
  diff(perc)*H}
```

This gives *Gamma* like biting weights, and when H is large, the variance of the biting weights converges to α. The default value was taken from [4]. We then compared this to the median and average of the stochastic simulation with 10,000 individuals, repeated 100 times. When plotted on the same graph, there was no visible difference between the lines for most values of *Pf*PR up to 75% (Figure 3). The numerical simulations for the ODEs were then calibrated to fit the exact solution. We found a good correspondence for $H = 20$ biting classes and for a maximum MOI of 300.

In all the simulations, the initial conditions were determined by the steady state, which can be solved analytically for the infinite-strain queuing models with homogeneous or heterogeneous biting. For the others, the simulations were run for 200 years, until the differences from the steady state were negligible.

Stochastic Ross-Macdonald Model We also formulated the analogous individual based model with H individuals, with individual biting weights ω_j. The initial MOI of the jth individual, denoted μ_j, was drawn from a Poisson distribu-

tion with mean $\omega_j h_0$. If $\mu_{j,t} > 0$, then we write that the person was infected and $x_{j,t} = 1$. Daily changes in MOI and the force of infection were simulated until the last infected person cleared the last parasite. As before, vectorial capacity was chosen to match the correct value $R_C = bcV(1+\alpha)/r$. The algorithm for simulating changes in MOI over time involved simulating lost infections and new infections each day, in three steps:

- Loss of infections: for every individual, $\mu_{j,t'} = \text{Binomial}[\mu_{j,t}, e^{-r}]$.
- Force of infection: compute $\tilde{X}_t = \sum_j c\omega_j x_j$, and $h_t = \dfrac{V\tilde{X}_t}{1+s\tilde{X}_t}$.
- New infections: $\mu_{j,t} = \mu_{j,t'} + \text{Poisson}[\omega_j h]$.

This is analogous to the dynamical equations, in the sense that it makes all of the same assumptions about the underlying process for individual humans.

In the case of homogeneous biting, the stochastic model is much simpler in that there is a single biting class.

Stage-Structured Infections In stage-structured models, the infections are subdivided into n distinct classes. Parasites can be lost from each stage at a stage-specific rate r_n, and they would pass from one stage to the next at a different stage-specific rate q_n. This gives a very flexible way of modeling any kind of structured loss from equations. To simulate stage-structured infections, we note that it is possible to formulate a deterministic model, but there would need to be an $n-$dimensional array of equations, because a person's infectious state would be described by the number of parasite types in each stage. For one solution to this problem, see [6].

Another solution is to set the stage-specific transition rates to $1/rn$ and then to assume that parasites all clear after passing from the lst class. This gives *Gamma* distributed waiting times, we also simulate individuals, but there are n distinct stages in an infection, and $x_j = 1$ if $\mu_{j,n} > 1$, for any n. The stochastic algorithm is:

- Advancing stages: for every individual and for all infection stages from $i = 1, 2, \ldots, n$,
  - $Q_{j,i,t} = \text{Binomial}[\mu_{j,i,t}, 1 - e^{-rn}]$.

- $\mu_{j,i,t'} = \mu_{j,i,t} - Q_{j,i,t} + Q_{j,i-1,t}$
- Note that $Q_{j,n,t}$ infections are lost.

- Force of infection: compute $\tilde{X}_t = \sum_j c x_j$, and $h_t = \dfrac{V \tilde{x}_t}{1 + s\tilde{X}_t}$.
- New infections are added at the first stage: $\mu_{j,1,t} = \mu_{j,1,t'} + \text{Poisson}[\omega_j h]$.

Solutions Our findings can be summarized in the following way:

1. For $\alpha = 4.2$, an exponential model that was fit to the exact solution (i.e. for completely interrupted transmission), predicted average waiting times to clear of 400 to 800 days, depending on baseline *Pf*PR (Figure 3).

2. The initial rate of decline was reasonably insensitive to R_C for values of R_C less than one (Figure 4). The asymptotic rate of decline was most sensitive to R_C, and this was usually reached when *Pf*PR reached 1%.

3. The initial rate of decline and MOI were most sensitive to α (Figure 5).

Empirical Estimates of Multiplicity of Infection These models consider the dynamics of malaria when a single person simultaneously carries multiple malaria phenotypes. This can occur for a variety of reasons such as:

1. One parasite "brood" emerges from the liver before other asexual blood stage parasite "broods" have cleared.

2. One person is bitten simultaenously by several infectious mosquitoes.

3. Each infectious bite transmits several different phenotypes.

Clearance occurs when none of the offspring of a brood remain.

The number of parasite broods is called the multiplicity of infection (MOI). The notion of a "brood" has never been given a clear operational definition in terms of parasite genetics. The problems of defining broods and relating these to empirical estimates of MOI are complicated by the genetic polymorphisms in virtually every gene involved with red blood cell invasion by merozoites, and by *var* gene expression while the parasite is inside the red blood cell. Thus, except in the case of selfing, most parasite broods are likely to differ at one or more

loci. Two genetically identical parasites, moreover, can have different phenotypes. The relevance of observed genetic diversity remains an open question. Here, we retain a vague definition of MOI, preferring the traditional notion of the number of distinct broods to any modern notion of the number of distinct genotypes. Improving the definition of MOI is beyond the scope of this modeling exercise.

Box #1: Parameters and Terms

- m: The ratio of mosquitoes to humans.
- g: The instantaneous death rate of adult *Anopheles* females. The average lifespan is $1/g$, and the probability of surviving one day is $p = e^{-g}$.
- f: Vector feeding rate. The average duration of the vector feeding cycle is $1/f$.
- Q: Human bloodmeals per bloodmeal
- a: Human feeding rate ($a = fQ$).
- τ: Number of days to complete sporogony
- c: Efficiency of transmission to mosquitoes, measured at low endemicity.
- b: Efficiency of transmission to humans.
- r: The waiting time to clear a simple, untreated infection, approximately 200 days.

Box #2: Malariometric Indices

- s: The stability index (fQ/g)
- $\sqrt{\alpha}$: Coefficient of variation of human exposure. We take $\alpha^2 = 4.2$. In the numerical simulations, $\alpha = \sum_j W_j(1-\omega_j)^2$.
- h: The force of infection. In these models $h = b\mathcal{E}$.
- $\tilde{X}$: Net human infectiousness, the probability a mosquito becomes infected after biting a human. Here, we use $\tilde{X} = \sum_j \omega_j W_j (1 - x_{0,j})$
- V: Vectorial capacity. $\lambda s^2 e^{-g\tau}$.
- $\mathcal{E}$: Entomological inoculation rate, $\mathcal{E} = V\tilde{X}/(1 + s\tilde{X})$.
- $\bar{X}$: Standard *Pf*PR at the steady state
- R_0: The basic reproductive number, $bcV(1+\alpha)/r$, where vectorial capacity is for a population with no vector control. In the Ross-Macdonald model, $\alpha = 0$.
- R_C: The controlled reproductive number, $bcV_C(1+\alpha)/r$, where vectorial capacity is for a population with a fixed level of vector control. In the Ross-Macdonald model, $\alpha = 0$.
- MOI: The multiplicity of infection

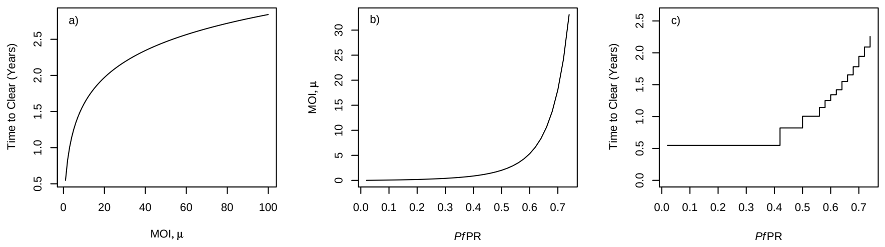

Figure 1: a) The expected waiting time to clear an infection, plotted as a function of MOI. The time lose one infection when MOI is μ is μ/r, and the expected waiting time to go from μ all the way down to zero is $1/r(1 + 1/2 + 1/3 + \ldots + 1/\mu)$. The waiting time is the sum of exponential distributions with different rate parameters, which does not have a simple closed-form expression. For $\mu = 100$ this is approximately 2.8 years, and for $\mu = 1,000$ it is approximately 4 years. b) For the infinite model with heterogeneous biting, we have plotted MOI as a function of *Pf*PR, c) For the same model, the average waiting time to clear as a function of baseline *Pf*PR.

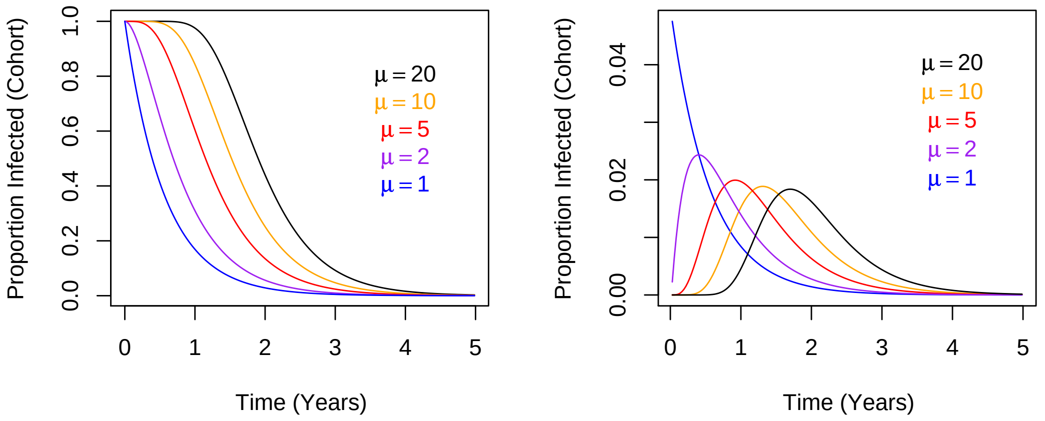

Figure 2: a) The proportion of a cohort that remains infected, starting with the specified MOI, and b) the proportion of the cohort that clear at a specific point in time.

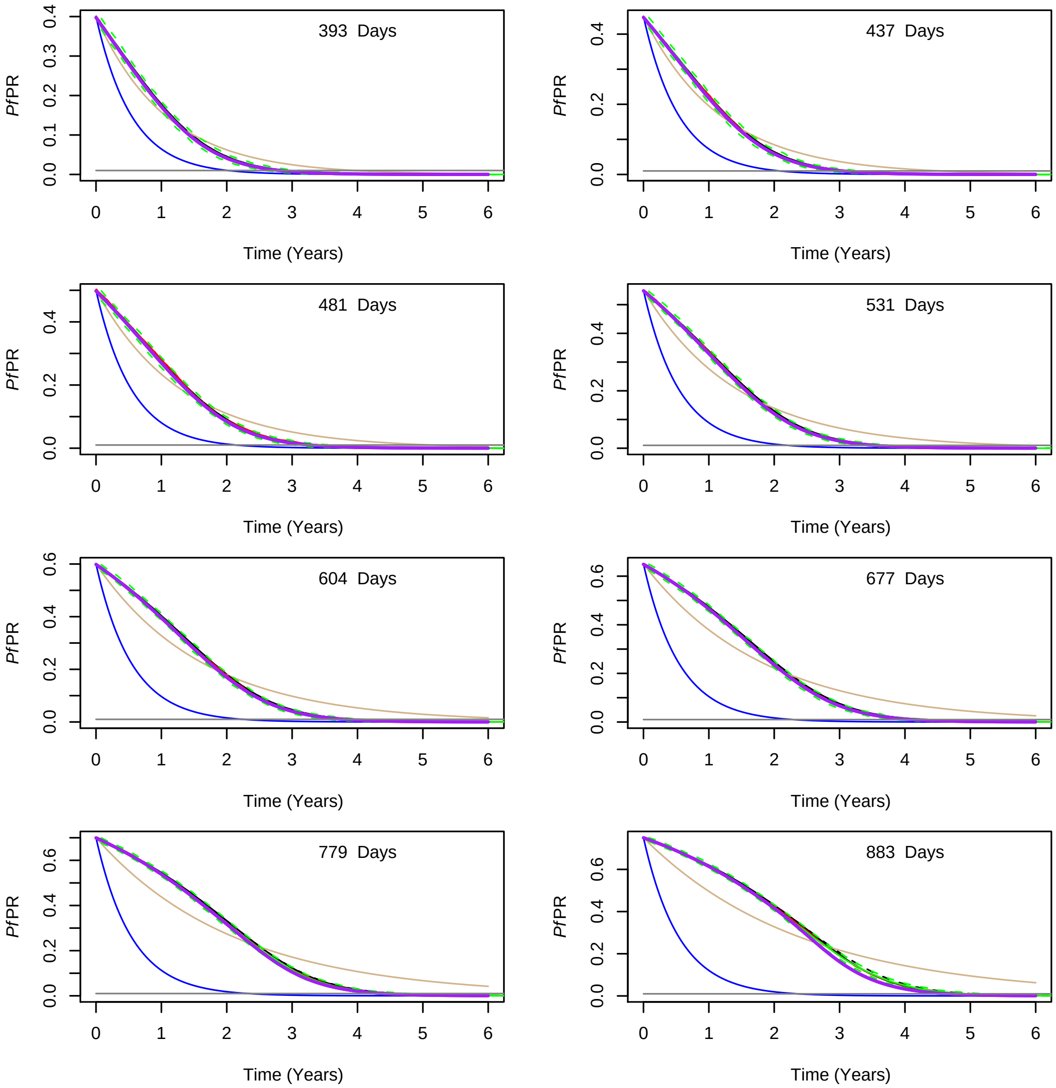

Figure 3: A comparison of the Ross-Macdonald model and a model with heterogeneous biting, infinite strains, and independent clearance for $R_C = 0$ for baseline *Pf*PR ranging from 0.4 up to 0.75. In each frame, an exact solutions with a maximum MOI of 300 is plotted in solid black, another exact solution with a maximum MOI of 1,000 is plotted in dashed black, numerical solutions to the ODEs are plotted in purple, the individual-based model with 10,000 individuals is plotted in red, green lines mark the the median (solid), 5th and 95th quantiles (dashed) from 100 realizations of the individual-based model with 1,000 individuals. All of these solutions lie on top of one another suggesting that the approximations that are introduced for numerical convenience introduce small and insignificant errors. The Macdonald model $e^{-t/200}$ is plotted in blue, and the exponential that gives the best-fit to the exact solution (by minimizing the sum of squared errors) is plotted in tan; the best-fit rate is reported on the graph.

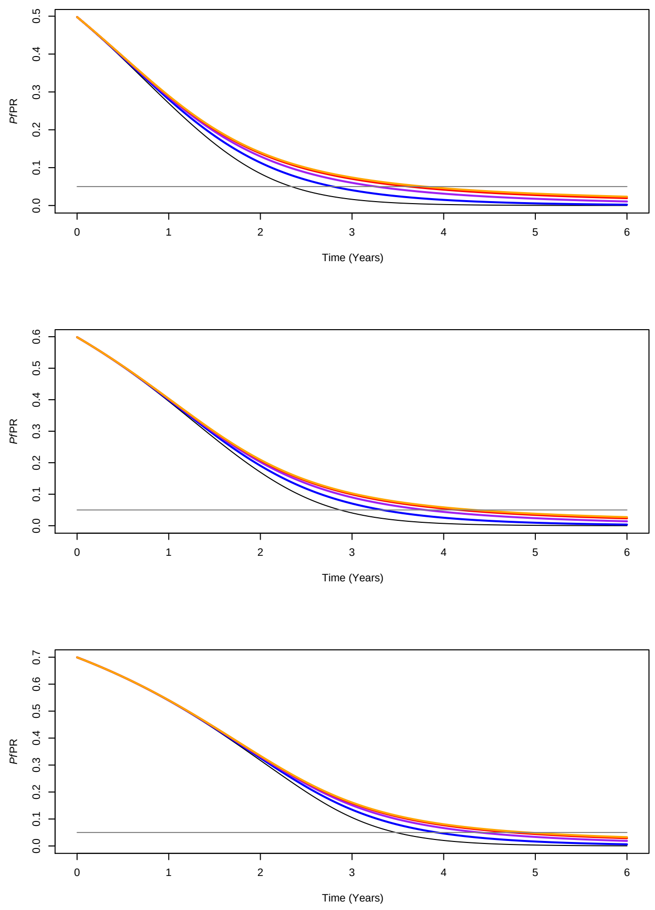

Figure 4: The decline in *Pf*PR is insensensitive to R_C initially, and only begins to make a difference as *Pf*PR approaches zero. This set of simulations uses the infinite strain model with independent clearance and heterogeneous biting. The colors represent $R_C = 0$ (black), 0.5 (blue), 0.8 (purple), 0.95 (red), and 1 (orange). The time to reach 5% differs by at most two years.

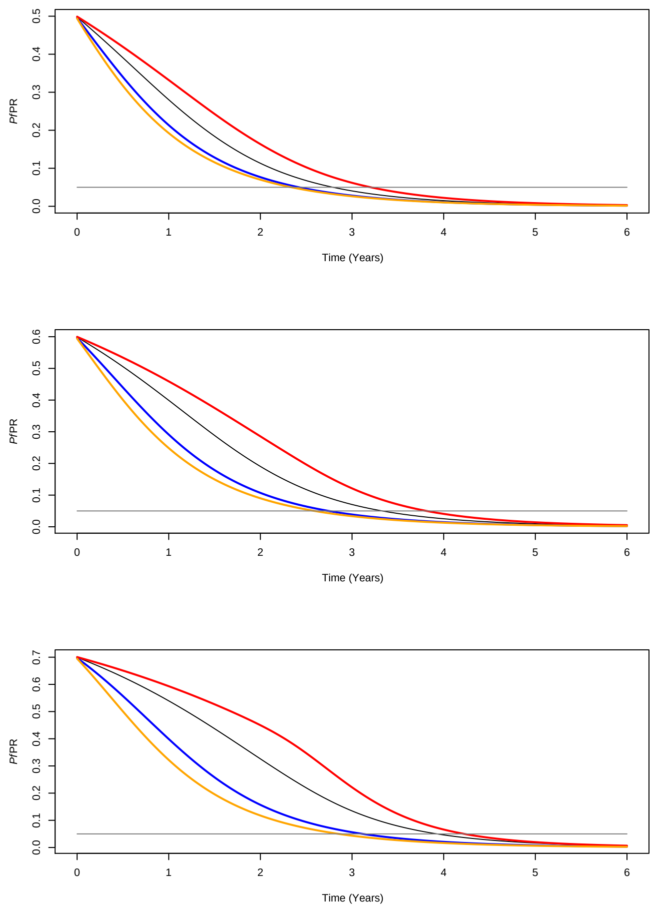

Figure 5: For the model with heterogeneous biting, infinite strains, and independent clearance. The rate of decline is sensitive to α, the index of biting disparity. For the same starting *Pf*PR, the baseline MOI increases with α. The values here are $\alpha = 6$ (red), $\alpha = 4.2$ (grey), $\alpha = 2$ (blue), $\alpha = 1$, and the GMEP model (yellow).

References

[1] D. L. Smith, F. E. McKenzie, *Malar J* **3**, 13 (2004).

[2] N. T. J. Bailey, *The Biomathematics of Malaria* (Oxford University Press, 1982).

[3] K. Dietz, L. Molineaux, A. Thomas, *Bull. Wld. Hlth. Org.* **50**, 347 (1974).

[4] D. L. Smith, J. Dushoff, R. W. Snow, S. I. Hay, *Nature* **438**, 492 (2005).

[5] G. Macdonald, G. W. Göeckel, *Bull World Health Organ* **31**, 365 (1964).

[6] L. C. Okell, C. J. Drakeley, T. Bousema, C. J. M. Whitty, A. C. Ghani, *PLoS Med* **5**, e226; discussion e226 (2008).
